# NEAT1 Promotes Epileptogenesis in Tuberous Sclerosis Complex

**DOI:** 10.1002/advs.202504316

**Published:** 2025-10-15

**Authors:** Suhui Kuang, Tinghong Liu, Zhirong Wei, Jinshan Xu, Jiayi Han, Jiaqi Wang, Feng Zhai, Yiran Tian, Shuli Liang

**Affiliations:** ^1^ Functional Neurosurgery Department National Children's Health Center of China Beijing Children's Hospital Capital Medical University Beijing 100045 China; ^2^ Key Laboratory of Major Diseases In Children Ministry of Education Beijing 100045 China; ^3^ Laboratory for Clinical Medicine Capital Medical University Beijing 100069 China

**Keywords:** epilepsy, epileptogenesis, LncRNA NEAT1, PI3K/AKT/mTOR, tuberous sclerosis complex

## Abstract

The primary neurological manifestations of tuberous sclerosis complex (TSC) are intractable epilepsy and intellectual impairment. Current treatment outcomes remain limited. Investigating the role of the epigenetic long non‐coding RNA NEAT1 in TSC‐related epilepsy and cognition is essential. RNA sequencing analysis of clinical tissue samples revealed that NEAT1 is differentially expressed in epileptogenic versus non‐epileptogenic tubers and enriched in the PI3K‐AKT signaling pathway. To prove and further investigate the functional role suggested by the earlier transcriptomic and pathway enrichment analyses, NEAT1‐overexpression and NEAT1‐knockdown TSC2 conditional knockout (CKO) mouse models, as well as TSC2‐KO cell models, are established. In vivo experiments demonstrated that NEAT1 knockdown reduced seizure frequency and improved spatial learning and working memory. Cellular analyses in TSC model further revealed that NEAT1 significantly regulates the PI3K/AKT/mTOR signaling pathway, neurotransmitter receptor balance, and outward potassium currents. Specifically, NEAT1 overexpression excessively activated the mTORC1 signaling, leading to changes in 4E‐BP1 and S6K and abnormal cell proliferation. Moreover, NEAT1 overexpression contributed to an imbalance in excitatory neurotransmitter receptors and outward potassium currents, resulting in neuronal hyperexcitability, whereas NEAT1 knockdown has the opposite effect. This study provides new insights into the transcriptional regulation of TSC‐related epilepsy, highlighting the therapeutic potential of NEAT1.

## Graphical abstract

The primary neurological manifestations of tuberous sclerosis complex (TSC) are intractable epilepsy and intellectual disability. NEAT1 is differentially expressed in TSC‐related epilepsy and influences neuronal excitability by regulating the PI3K/AKT/mTOR signaling pathway. Knockdown of NEAT1 reduces seizure frequency and improves cognitive function, suggesting its therapeutic potential and providing a new direction for personalized treatment of TSC.

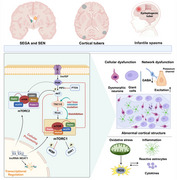

## 1 Introduction

**Figure 1:**
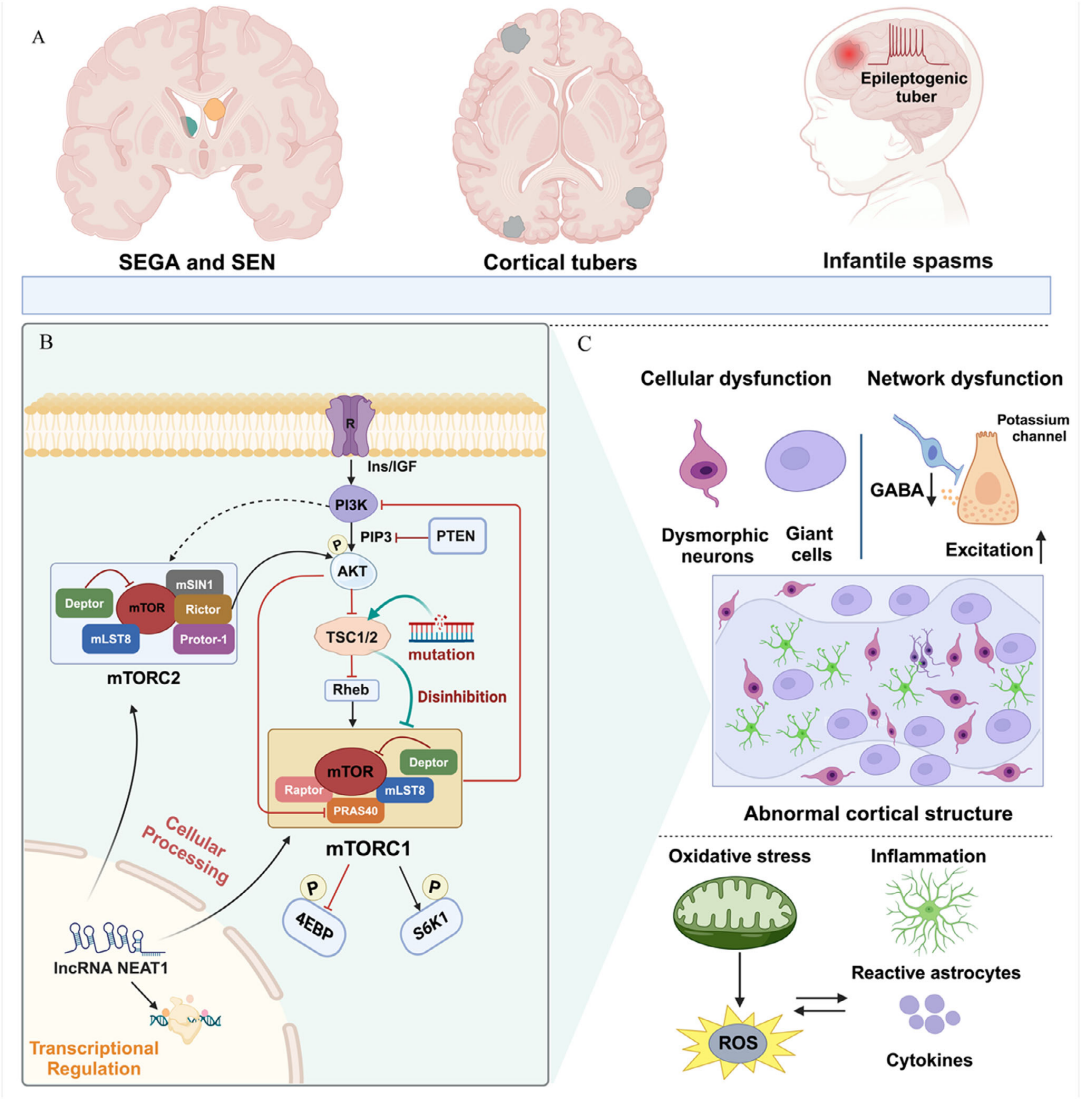
A diagram of the potential neuropathology and mechanisms of TSC‐related epilepsy. A) Excessive activation of mTOR pathway causes excessive cell growth, abnormal protein synthesis, and neuronal excitability, contributing to the formation of epileptogenic tubers, subependymal giant cell astrocytomas (SEGA), and subependymal nodules (SENs), which are hallmark features of TSC. B) The PI3K‐AKT‐TSC1/TSC2 complex‐mTORC1‐4EBP1/S6K pathway plays a critical role in regulating cell growth, metabolism, and survival, and its dysregulation is central to the pathogenesis of tuberous sclerosis complex (TSC). This pathway is initiated by PI3K activation through upstream signals like IGF, which activates AKT by PIP3. Once activated, AKT phosphorylates and inactivates the TSC1/TSC2 complex, which normally inhibits Rheb, a small GTPase that activates mTORC1. As a result, mTORC1 is activated and phosphorylates key downstream targets like 4EBP1 and S6K, which promote protein synthesis, cell growth, and metabolic processes. Additionally, mTORC1 regulates the PI3K/AKT pathway through negative feedback mechanisms. In TSC, mutations in the TSC1 or TSC2 genes impair the TSC complex's function, leading to uncontrolled mTORC1 activation. C) The underlying epileptogenic mechanisms involve neuronal dysfunction resulting from the cortical dyslamination in epileptogenic tubers, as well as the disruption of neurotransmitter receptor function and potassium ion channel imbalance, which leads to excessive neuronal excitability and seizures. Additionally, oxidative stress and inflammation further contribute to neuronal damage and excitability in these areas.

Tuberous sclerosis complex (TSC) is a multi‐system autosomal dominant genetic disorder that is caused by pathogenetic variants *TSC1* and *TSC2* genes, leading to overactivation of the mTOR pathway.^[^ ^1^, ^2^, ^3^, ^4^ ^]^ The disorder is primarily characterized by the development of benign tumors and pathological lesions in multiple organs.^[^ ^1^, ^5^, ^6^ ^]^ The most common neurological manifestations include drug‐resistant epilepsy, intellectual disability, and autism spectrum disorder.^[^ ^1^, ^3^, ^7^, ^8^, ^9^ ^]^ Even so, 80% ‐90% of patients with TSC still develop epilepsy, which often begins with infantile spasms and develops drug resistant epilepsy (DRE) in two‐thirds of cases.^[^ ^10^, ^11^ ^]^ Cortical tubers, subependymal nodules, and subependymal giant cell astrocytoma (SEGA) are the main neuropathological lesions in the brains of patients with TSC.^[^ ^1^, ^2^ ^]^ Cortical tubers represent areas of disrupted cortical stratification containing abnormal cell types, including dysmorphic neurons, giant cells, and reactive astrocytes, which are classified as epileptogenic tubers (ETs) and non‐epileptogenic tubers (Non‐ETs).^[^ ^2^, ^12^, ^13^, ^14^ ^]^ Epileptogenic tubers are considered the primary neuropathological substrates of epilepsy in patients with TSC (**Figure** **1**).^[^ ^2^, ^12^ ^]^ In addition, Epidemiological studies have shown no significant sex‐related differences in the incidence of TSC.^[^ ^15^ ^]^

Over the past decade, there has been repeatedly demonstrated that an aberrant activation of the mTOR signaling cascade may represent a pathogenic mechanism in TSC‐related epilepsy.^[^ ^2^, ^12^ ^]^ mTOR is a serine–threonine protein kinase that acts through different protein complexes of mTORC1 or mTORC2.^[^ ^2^ ^]^ These protein complexes are distinguished by composition and function.^[^ ^2^, ^12^, ^16^ ^]^ The mTOR signaling pathway exerts a crucial role during the development of the cerebral cortex. The excessive mTORC1 activation caused by loss‐of‐function of either *TSC1* or *TSC2* gene mutation can result in abnormal development of the cortex with alterations in neuronal and glial differentiation, neural cell size, axon and dendrite growth, and synaptogenesis.^[^ ^1^, ^2^, ^17^ ^]^ The excessive activation of mTORC1 has been reported as the leading cause of TSC‐related epilepsy, and mTOR inhibitors can decrease the development of seizures by targeting mTORC1 (Figure 1).^[^ ^8^, ^18^ ^]^ The role of mTORC2 has been less explored. mTOR inhibitors are the first available therapy targeting the pathogenic mechanism of TSC.^[^ ^19^ ^]^ However, not all patients with TSC‐related epilepsy respond to mTOR inhibitors.^[^ ^3^, ^8^ ^]^ More than 60% of patients fail to achieve seizure freedom, experiencing only partial reductions in seizure frequency, which are very similar to the classical anti‐seizure medications.^[^ ^20^, ^21^ ^]^ Thus, the phenotypes of TSC‐related epilepsy should not be exclusively attributed to mTORC1 hyperactivity, and current understanding of the molecular mechanism in TSC‐related epilepsy remains incomplete.^[^ ^8^ ^]^

Development of further prognostic biomarkers and the identification of novel antiepileptic targets depend on elucidating the underlying genetic and phenotypic heterogeneity. Long non‐coding RNAs (LncRNAs) are involved in the complex epigenetic regulation of epilepsy, which are related to retinal neurotransmission, impairment of neurons, cellular signaling pathway, and synaptic remodeling.^[^ ^22^ ^]^ LncRNA NEAT1 (collectively referred to as NEAT1) has been confirmed to be involved in many diseases and be a key regulatory gene in regulating cell differentiation and growth, which is highly expressed in the brain.^[^ ^23^ ^]^ In the cortical tissue samples of patients with temporal lobe drug‐resistant epilepsy, as well as in CTX‐TNA astrocytes from epileptic model rats, overexpression of NEAT1 significantly upregulated the expression of IL‐1β, IL‐6, cyclooxygenase‐2 (COX‐2), TNF‐α, and Notch1, suggesting that NEAT1 may be involved in the development and progression of epilepsy by regulating neuroinflammatory responses.^[^ ^24^ ^]^ In addition, NEAT1 can function as a regulatory factor within PI3K‐AKT‐mTOR signaling pathway and even regulate downstream signaling molecules, which are pivotal to TSC‐related epilepsy (Figure 1).^[^ ^23^, ^25^ ^]^ Therefore, our research team is dedicated to investigating the role of NEAT1 within the mTOR signaling pathway in TSC‐related epilepsy. Furthermore, this study aims to advance the understanding of the underlying epigenetic mechanisms and provide a foundation for the development of novel molecular targets and therapeutic strategies in TSC‐related epilepsy.

## 2 Methods

### 2.1 Cortical Tissues

The cortical tubers were resected surgically in patients with TSC from Beijing Children's Hospital, including 15 pairs of ETs and non‐ETs (**Table** **1**). The inclusion criteria of patients for this study were as follows: patients diagnosed with TSC based on clinical diagnostic criteria with *TSC1* or *TSC2* genes mutation^[^ ^26^ ^]^; All subjects were diagnosed with drug‐resistant epilepsy that was unresponsive to at least two types of anti‐seizure medications; Stereoelectroencephalography (SEEG) monitoring was conducted to locate ET and non‐ETs in patients who underwent resective surgery; ETs and adjacent non‐ETs were removed under intraoperative neuroimaging guidance; Currently, no definitive biomarkers or imaging criteria exist to confirm whether a resected tuber is epileptogenic. Therefore, all participants were seizure‐free for at least one year postoperatively. Additionally, the accurate resection for both ETs and adjacent non‐ETs was confirmed by postoperative MRI. Seizure outcomes were evaluated according to International League Against Epilepsy (ILAE) outcome classification: ILAE type 1 (seizure‐free) and ILAE type 2–6 (not seizure‐free). These procedures were performed to avoid misidentifying epileptogenic tubers as non‐epileptogenic ones and subsequently using them for experimental research. Tissue collection and manipulation were authorized by the Ethics Committee of Beijing Children's Hospital. Informed consents were collected from caregivers of all subjects.

**Table 1:** Clinical characteristics of the patients with TSC‐related epilepsy and one‐year follow‐up results.

	Age (years)	Gender	Onset of epilepsy (months)	Seizure type at onset	Preoperative seizure frequency (per week)	postoperative seizure control	Gene mutation
**Patient‐1**	10.8	Female	6.0	Epileptic spasm	>10	Seizure freedom	unidentified
**Patient‐2**	12.8	Female	36.0	Focal cognitive seizure with impaired awareness	4‐10	Seizure freedom	*TSC1*
**Patient‐3**	9.8	Male	84.0	Focal cognitive seizure	1‐3	Seizure freedom	unidentified
**Patient‐4**	15.0	Male	170.0	Focal clonic seizure with impaired awareness	>10	Seizure freedom	*TSC2*
**Patient‐5**	3.4	Male	4.0	Focal clonic seizure with impaired awareness	>10	Seizure freedom	unidentified
**Patient‐6**	5.1	Female	28.0	Epileptic spasm	>10	Seizure freedom	*TSC2*
**Patient‐7**	5.0	Male	16.0	Focal clonic seizure with impaired awareness	>10	Seizure freedom	*TSC2*
**Patient‐8**	8.1	Male	78.0	Focal clonic seizure	>10	Seizure freedom	*TSC1*
**Patient‐9**	12.7	Male	18.0	Focal clonic seizure	>10	Seizure freedom	*TSC1*
**Patient‐10**	4.8	Male	28.0	Epileptic spasm	>10	Seizure freedom	*TSC2*
**Patient‐11**	9.6	Female	31.6	Focal clonic seizure	1‐3	Seizure freedom	unidentified
**Patient‐12**	3.9	Female	16.8	Focal emotion seizure with impaired awareness	>10	Seizure freedom	*TSC2*
**Patient‐13**	6.2	Female	27.2	Behavioral arrest seizure	>10	Seizure freedom	unidentified
**Patient‐14**	6.8	Male	14.0	Epileptic spasm	>10	Seizure freedom	*TSC1*
**Patient‐15**	10.2	Male	48.0	Focal clonic seizure	>10	Seizure freedom	unidentified

### 2.2 RNA‐Sequencing

Total RNA was extracted from brain tissue using the QIAGEN RNA extraction kit, following the manufacturer's protocol. RNA‐Seq libraries were constructed using the KAPA RNA‐Seq Library Preparation Kit. High‐throughput sequencing was performed on the Illumina HiSeq platform using the PE150 kit. The sequencing depth exceeded 30 million reads per sample, ensuring sufficient data for downstream analyses. The sequencing data generated from the Illumina platform were in BCL format. It was subsequently converted to FASTQ format (version: Illumina 1.8+) using the bcl2fastq software (v2.17.1.14, Illumina, Inc.). Data quality control was performed using Trimmomatic software to remove low‐quality reads and adaptors. After quality control, the data were compared to the reference transcriptome using STAR (version 2.5.3a). The study of differentially regulated gene expression (Genes with a fold change greater than 1.5 and a p‐value less than 0.05 are considered significantly differentially expressed) was conducted using DESeq2 in R (version 4.4.0), using three biological replicates. Kyoto Encyclopedia of Genes and Genomes (KEGG) enrichment analysis of differentially expressed genes was performed using the ClusterProfiler R package.

### 2.3 Animals

TSC2 flox/flox‐GFAP‐Cre knockout (TSC2‐GFAP‐CKO) mice, with a C57BL/6 genetic background, were conditionally inactivated the TSC2 gene in glia and neurons driven by a glial fibrillary acidic protein (GFAP) promoter through the Cre‐LoxP system, as described previously.^[^ ^27^, ^28^ ^]^ Genotyping was performed during the first week of life to verify the presence of the intended genetic alterations. The mice were aged between 6 to 8 weeks and weighed between 18 to 30 grams with no restriction on sex. The animals were kept in a regulated environment with a temperature of 22±2 °C, humidity maintained between 40–60%, and a 12‐h light/dark cycle. They were given ample food and water to support their proper development and well‐being. All procedures were conducted in accordance with institutional and national guidelines for the care and use of laboratory animals. Group allocation was performed using stratified randomization based on baseline characteristics, ensuring balanced distribution across groups. Experimenters involved in outcome measurement and statistical analysis were fully blinded to treatment conditions throughout the study.

An epileptic mouse model was induced with kainic acid (Sigma–Aldrich, USA) at a dose of 5 mg/kg via intraperitoneal injection for 14 consecutive days. Mice that exhibited Racine stage IV‐V were considered valid models. Tuberous sclerosis complex‐related epilepsy in patients includes both focal and generalized seizure types, and KA‐induced seizures in mice also encompass a spectrum of seizure manifestations, including focal onset and secondary generalization. Mice were randomly assigned into four groups (n = 6): Control group: Wild‐type (WT) mice without any viral injection, used as baseline controls. Model control group: TSC2‐GFAP‐CKO mice that did not receive any viral treatment, representing the TSC model. Model NEAT1 group: TSC2‐GFAP‐CKO mice injected with NEAT1‐overexpression lentivirus via stereotactic delivery. Model siNEAT1 group: TSC2‐GFAP‐CKO mice injected with NEAT1‐targeting siRNA lentivirus (siNEAT1) via stereotactic delivery. The lentiviral vectors for NEAT1 overexpression and NEAT1 knockdown were commercially synthesized and provided by GenePharma (Shanghai, China).

Stereotaxic injection into the lateral ventricle: mice were initially anesthetized with isopentobarbital (50 mg/kg body weight) and secure it in a stereotaxic frame. The surgical area was cleaned, and a small incision was made on the scalp. A fine dental drill was used to create a hole at the designated coordinates for the lateral ventricle. The injection needle was inserted into one lateral ventricle of the brain at the following coordinates: 0.2 mm posterior, 1.0 mm lateral, and 2.0 mm deep. NEAT1 lentivirus (5 µL) and Entranster‐in vivo transfection reagent (2.5 µL) (Engreen Biosystem, Co., Ltd.) were slowly injected according to the manufacturer's instructions. The needle was left in place for a short period before being removed. The incision was closed with sutures or tissue adhesive, and the mouse was placed on a heating pad to aid recovery. Post‐surgical monitoring for complications was performed, and pain relief was provided as necessary.

EEG monitoring: Following anesthesia, a 0.5 mm screw was implanted 2 mm anterior and 2 mm lateral to the anterior fontanelle, serving as both the reference and ground electrode. A 0.8 mm screw was placed 2 mm posterior and 2 mm lateral to the anterior fontanelle, reaching the cortex, and functioned as the recording electrode. Postoperative care included daily injections of 2000 IU of penicillin for the first three days to prevent infection. Seven days after the implantation, the mice were secured in a customized plastic fixator, and EEG recordings were performed while they were fully awake. To minimize time‐related variability, EEG recordings were conducted between 9:00 and 11:00 AM. Epileptic discharges were defined as abnormal EEG activity, while seizure events were identified by continuous, synchronous, explosive electrical activity exceeding 2 Hz and lasting for at least 3 s on EEG. The EEG analysis system was NicoleOne nEEG Module Reader Version 5.22.1046.

### 2.4 Morris Water Maze Tests

The Morris water maze apparatus featured a circular pool with a diameter of 1.5 meters, divided into four quadrants (NE, SW, SE, and NW). The pool was surrounded by light blue curtains. The water level was maintained at a depth of 27 cm, and the water was mixed with milk powder to eliminate visual cues. The temperature was controlled between 23 °C‐24 °C. A hidden platform with a diameter of 12 cm was submerged 1–2 cm below the water surface. The experimental room was soundproof and free of direct lighting. The experimental room was soundproof and free of direct lighting. The experimenters conducting the behavioral scoring were blinded to group assignments.

Experient 1: The mice underwent a one‐day pretraining session. Experiment 1 consisted of two phases: acquisition trial and probe trial. During the acquisition trial, the mice were initially placed on the platform in the SW quadrant for 15 s and then randomly released from the remaining three quadrants (NW, SW, NE) with their head facing the tank wall. Each trial lasted for 60 s, and their activities were recorded. If the mice failed to locate the platform within 60 s, they were gently guided to it and allowed to stay there for 15 s (this adaptation training was performed only once). Three trials were conducted per day over three consecutive days, resulting in a total of nine trials per mouse. In the probe trial, conducted 24 h after the acquisition phase, the platform was removed, and the release point remained the same as in the acquisition trial. The number of times the mice crossed the platform's location within 60 s was recorded, along with the swimming time and path.

Experiment 2: The water maze apparatus and experimental setup were identical to those used in experiment 1. Before the official testing, the rats underwent a one‐day pretraining session. Each rat completed four pairs of trials per day for four consecutive days. The platform and entry point locations were randomly changed between each pair of trials. Each pair consisted of Trial 1 and Trial 2. In Trial 1, the rats were placed on the platform for 5 s before being released into the water, facing the tank wall. They were given 60 s to locate the platform. If they failed to find it within this time, they were gently guided to the platform and allowed to remain there for 30 s (this adaptation training was performed only once). Following a 15‐s intertrial interval, Trial 2 began. The rats were released into the water under the same conditions as in Trial 1, with identical platform and entry point locations. The swimming time and path were recorded for both trials.

### 2.5 RNA Immunoprecipitation‐qPCR

Tissues were homogenized in RIP lysis buffer, centrifuged, and the supernatant collected. After reserving input samples, lysates were incubated with anti‐PI3K (Abcam, 1:50), anti‐AKT(CST 1:50), anti‐mTOR antibodies(Abcam, 1:50), or IgG control overnight at 4 °C, followed by incubation with Protein A+G agarose beads. Beads were washed, and RNA‐protein complexes were eluted at 55 °C. Total RNA was purified using the RNAeasy kit (Beyotime, China) and reverse transcribed using the PrimeScript RT reagent Kit (Takara, Japan). NEAT1 enrichment was analyzed by qRT‐PCR, normalized to input, to assess its interaction with PI3K, AKT, and mTOR pathway proteins. Experimenters involved in outcome measurement and statistical analysis were fully blinded to treatment conditions throughout the study. Three independent biological replicates were used for the experiments.

### 2.6 Cell Culture and Transfection

TSC2‐KO 3T3 cells were kindly provided by Professor Yushi Zhang from Peking Union Medical College Hospital. The cell culture procedure was described as follows: First, DMEM (Corning, USA) containing FBS (Hyclone, Australia) and 100 U mL^−1^ penicillin and 100 µg mL^−1^ streptomycin (Pricella, China) was added to six‐well plates, and then the cells were seeded into the six‐well plates at a predetermined density. Second, the cells were cultured at 37 °C in a 5% CO_2_ incubator and subcultured in accordance with their growth. In this study, the NEAT1 lentivirus and negative controls (scramble) constructs were obtained from GenePharma (Shanghai, China). Cell transfection was performed using NEAT1 lentivirus and HiTransG A transfection reagent (GenePharma, Shanghai, China). After 48 h, stably transfected cells were selected using puromycin. The cells were grouped into four types: control group, model control group (TSC2‐KO cell), model NEAT1 group, and model siNEAT1 group.

### 2.7 RT‐qPCR

The total RNA from cells and tissues was extracted by TRIzol reagent (Invitrogen, USA). RNA concentration and purity were assessed via ultraviolet (UV) absorption with a NanoDropND‐2000 (Thermo Fisher Scientific, USA), with DEPC‐treated water as the baseline reference.Single‐stranded complementary DNA (cDNA) was synthesized using PrimeScriptTM Master Mix(Takara, Japan), following the manufacturer's protocol. RT‐qPCR was performed using SYBR‐Green‐Master Mix (Thermo Fisher Scientific, USA) on the Applied Biosystems QuantStudio 6 system. The primers used in this study were synthesized by Beijing Liuhe BGI Co.,Ltd. and were listed as follows: NEAT1: forward, 5′‐GGAGCCAACCTGCCCTGAAT‐3′, reverse, 5′‐CCACAGGCTACCCTCTGCTC‐3′; NMDA receptor: forward, 5′‐CTGCGACCCCAAGATTG TCAA‐3′, reverse, 5′‐ TATTGGCCTGGTTTACTGCCT‐3′; GABA‐A receptor: forward, 5′‐TGG AGGGTTGGACCTCATGTT‐3′, reverse, 5′‐ GATTCAGGCGGAGTTAGAGGC ‐3′; β‐actin: forward, 5′‐ GGCTGTATTCCCCTCCATCG ‐3′, reverse, 5′ – CCAGTTGGTAACAATGCCATGT ‐3′. The gene expression level was normalized to β‐actin expression and calculated using the 2^^–ΔΔCt^ method. Experimenters involved in outcome measurement and statistical analysis were fully blinded to treatment conditions throughout the study. Three independent biological replicates were used for the experiments.

### 2.8 Western Blotting

Total protein was extracted from cells and tissues, and the protein concentration was measured using the BCA protein concentration assay kit (Thermo Fisher Scientific, USA). Proteins were separated by sodium dodecyl sulfate‐polyacrylamide gel electrophoresis (SDS‐PAGE) and transferred onto polyvinylidene difluoride (PVDF) membranes (Millipore, USA). The PVDF membranes were then blocked with 5% non‐fat milk for 2 h at room temperature to reduce non‐specific binding. After blocking, the membranes were incubated overnight at 4 °C with primary antibodies diluted in TBST: β‐actin (1:10 000, Proteintech), NMDA receptor (1:1000, Abcam,), GABA receptor (1:1000, Abcam), PI3K (1:1000, Proteintech), AKT (1:2000, Proteintech), AKT (phospho T308) (1:500, Bioss), AKT (phospho T473) (1:2000, Proteintech), mTOR (1:5000, CST) and mTOR (phospho S2448) (1:5000, CST), S6K1 (1:500, Bioss), phospho‐S6K1 (1:500, Bioss), 4E‐BP1 (1:500, Bioss), phosphor‐4E‐BP1 (1:500, Bioss). Next, the membranes were washed three times with TBST for 5 min each, followed by incubation with horseradish peroxidase‐conjugated goat anti‐rabbit IgG and horseradish peroxidase‐conjugated goat anti‐mouse (1:1000, Biyuntian Biotechnology) for 1 h at room temperature. Afterward, the membranes were washed three times with TBST for 5 min each. Finally, an enhanced chemiluminescent substrate was applied to the membranes to visualize the protein bands, and protein expression levels were quantified by ImageJ software (NIH, USA). Experimenters involved in outcome measurement and statistical analysis were fully blinded to treatment conditions throughout the study. Three independent biological replicates were used for the experiments.

### 2.9 MTT Assay

The four types of cells were seeded into 96‐well plates at a density of 1 × 10^4^ well for 24 and 48 h. The TSC2‐KO cells were exposed to different concentrations of mTOR inhibitor (Selleck, USA) (1‐100 nm) for 48 h and NEAT lentiviral transfection for 24 or 48 h. MTT assay was performed to assess cell viability and proliferation by measuring absorbance at a wavelength 490 nm using a Microplate Reader (Thermo Fisher Scientific, USA). Experimenters involved in outcome measurement and statistical analysis were fully blinded to treatment conditions throughout the study. Three independent biological replicates were used for the experiments.

### 2.10 Patch Clamp

The patch‐clamp technique was used for recording potassium currents. The extracellular solution contained (in mM): 137 NaCl, 4 KCl, 2 CaCl_2_, 1 MgCl_2_, 10 glucose, 10 HEPES with the pH adjusted to 7.4 using NaOH. The intracellular solution consisted of (in mM): 4 NaCl, 140 K‐Gluconate, 0.1 CaCl_2_, 1 MgCl_2_, 2 Mg‐ATP, 10 HEPES, 1 EGTA with the pH adjusted to 7.2 using KOH. Glass micropipettes were pulled using a Sutter P‐1000 and filled with extracellular solution. The electrodes were then used to approach individual cells immersed in the extracellular solution under a micromanipulator (Scientifica, US). A high‐resistance giga‐seal (>1 GΩ) was formed by monitoring resistance changes and applying gentle suction. Once the seal was established, additional suction was applied to rupture the membrane and achieve the whole‐cell configuration. Voltage‐clamp recordings were conducted by holding the cell membrane potential at −80 mV, followed by step voltage pulses ranging from −70 mV to +70 mV in 10 mV increments. Each voltage step was applied for 300 ms, with a 2‐s interval between sweeps. Data quality was ensured by maintaining the following criteria: seal resistance >1 GΩ, leak current <100 pA, series resistance <20 MΩ, and membrane resistance >200 MΩ. Experimenters involved in outcome measurement and statistical analysis were fully blinded to treatment conditions throughout the study. Three independent biological replicates were used for the experiments.

### 2.11 Statistical Analysis

All statistical analyses were performed using Prism 10.0 (GraphPad Software) and R language (Version 4.4.0, R Foundation for Statistical Computing, Vienna, Austria), respectively. Normality of the data was first tested using the Shapiro‐Wilk test. Normally distributed data were analyzed using one‐way analysis of variance (ANOVA) followed by the Bonferroni correction for multiple groups and the Student t‐test for two groups. Non‐normally distributed data were analyzed using the Kruskal–Wallis test. Quantitative data are expressed as mean ± standard error of the mean (SEM). Statistical significance was defined as *p* value<0.05.

## 3 Result

### 3.1 Transcriptomic Analysis Revealed NEAT1 Differential Expression

**Figure 2:**
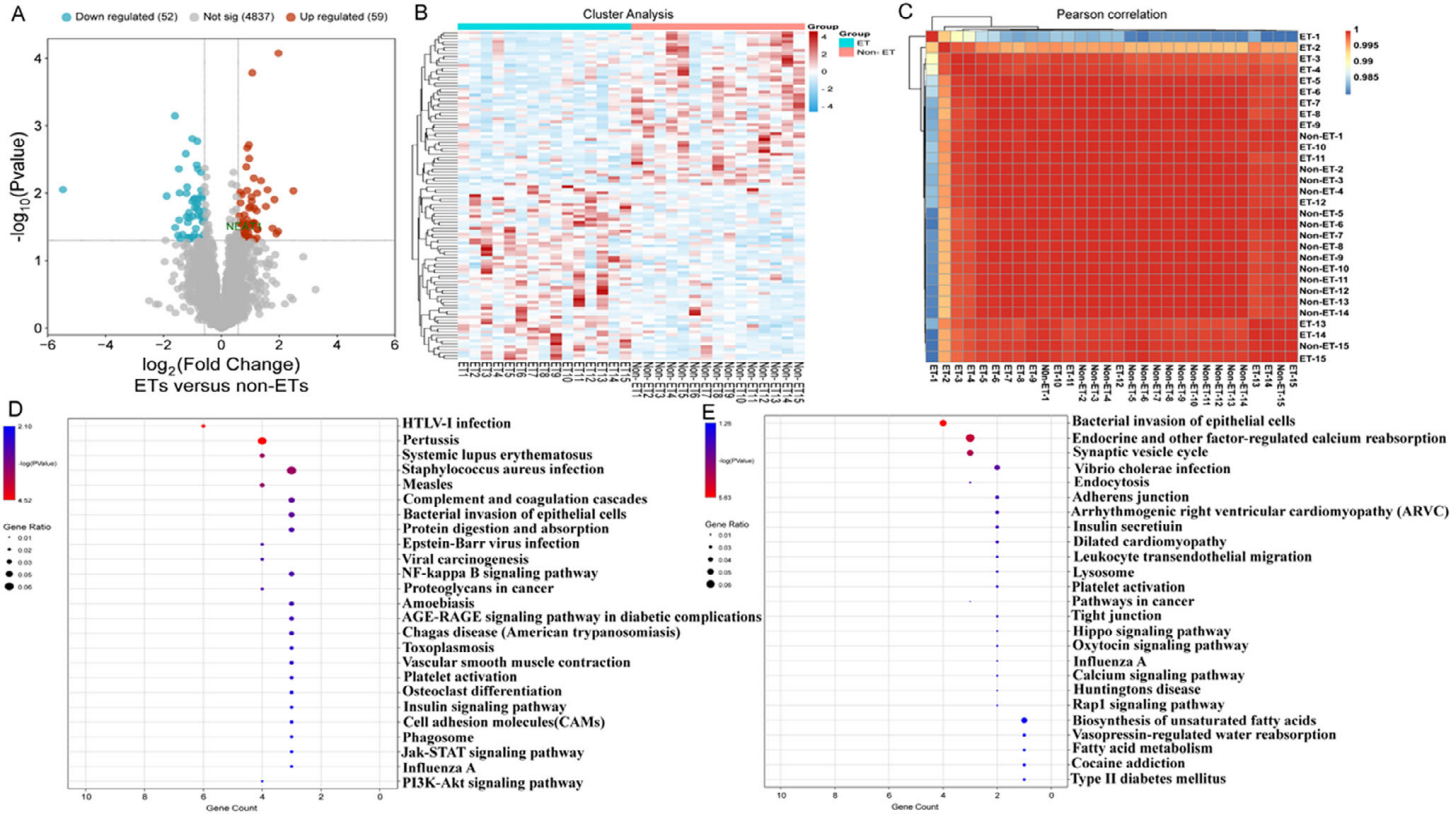
The visual images of DEGs expression in TSC‐related epilepsy transcriptomic profile. The visual images of DEGs expression in TSC‐related epilepsy transcriptomic profile. A) Differential expression analysis revealed a total of 111 differentially expressed genes between ETs and non‐ETs, comprising 59 upregulated genes and 52 downregulated genes. NEAT1 was highly expressed in ETs, n = 15. B) The LncRNA data of samples were subjected to clustering analysis using complete linkage. The clustering analysis was performed to identify co‐expression patterns with biological significance and reliability. The ETs and non‐ETs were distinctly clustered and distinct gene expression profiles between the two groups. Samples within each group exhibited high intragroup similarity, reflecting consistent expression patterns among replicates, n = 15. C) The correlation between biological replicates have an R^2^ > 0.8, which selected samples meet the required criteria, n = 15. D) The GO and KEGG enrichment analysis revealed that the upregulated genes were predominantly involved in immune and inflammatory processes, n = 15. E) The GO and KEGG enrichment analysis revealed that the downregulated genes were mainly enriched in the synaptic vesicle cycle and ion channels, n = 15. Gene counts were modeled using a negative binomial distribution, and statistical significance was assessed using the Wald test. Pearson's Correlation Coefficient (R^2^) is typically used to assess the correlation between biological replicate samples. ^*^ *P* < 0.05.

Significant differences in gene expression between ETs and non‐ETs were identified using the limma package in R. Differential expression analysis revealed a total of 111 differentially expressed genes between ETs and non‐ETs, comprising 59 upregulated genes and 52 downregulated genes (**Figure** **2**A). Notably, NEAT1 was significantly upregulated in ETs. The clustering analysis was performed to identify co‐expression patterns with biological significance and reliability. The ETs and non‐ETs were distinctly clustered, and distinct gene expression profiles between the two groups (Figure 2B). Samples within each group exhibited high intragroup similarity, reflecting consistent expression patterns among replicates (Figure 2B,C). Gene Ontology (GO) and Kyoto Encyclopedia of Genes and Genomes (KEGG) enrichment analyses were conducted to further explore the potential biological functions of differentially expressed genes. The GO and KEGG enrichment analysis revealed that the upregulated genes were predominantly involved in immune and inflammatory processes, while the downregulated genes were mainly enriched in the synaptic vesicle cycle and ion channels (Figure 2D,E). The majority of upregulated gene sets were involved in immuno‐inflammatory responses, including cytokine‐cytokine signaling pathways. Moreover, The PI3K‐AKT pathway, known for its key regulatory role in TSC‐related epilepsy, was also enriched.

### 3.2 NEAT1 Regulates Seizures and Excitability in TSC2‐CKO Animal Model

**Figure 3:**
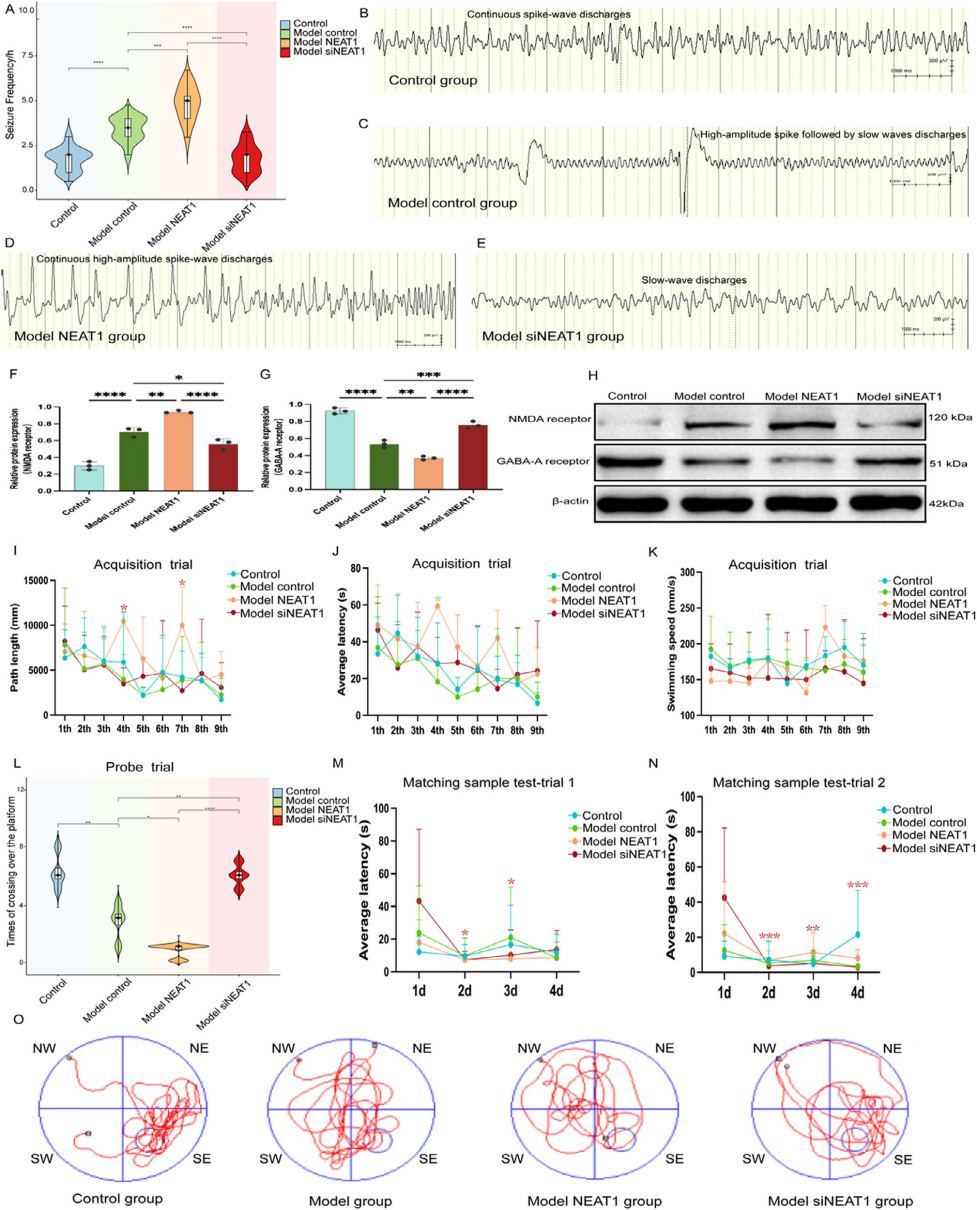
LncRNA NEAT1 regulates the seizure frequency and cognition. A) Mice in the model control group exhibited a significantly higher seizure frequency compared to the control group (P < 0.0001). The seizure frequency of mice in model NEAT1 group was significantly higher than that in model control group (P = 0.0002), while the model siNEAT1 group showed a significant reduction in seizure frequency (P < 0.0001) than that in model control group, n = 6. B) The electroencephalogram (EEG) of control group mice during epileptic seizures showed continuous spike‐wave discharges. Seizure type: clonic seizure. C) The EEG of model control group mice during brief spasm seizures showed characteristic patterns of activity. Seizure type: spasm seizure. D) The EEG of the model NEAT group mice during epileptic seizures showed continuous high‐amplitude spike‐wave discharges. Seizure type: tonic‐clonic seizures. E) The EEG of the model siNEAT group mice during epileptic seizures showed slow‐wave discharges and intermittent spike‐wave discharges. Seizure type: head nodding seizure. F‐H): The protein expression of NMDA receptors (P < 0.0001) was higher in TSC2‐CKO mice compared to wild‐type mice, while GABA‐A receptor (P < 0.0001) expression was reduced. In the model NEAT1 group, NMDA receptor expression was significantly elevated (P = 0.0017), while GABA‐A receptor expression was reduced (P = 0.0026) compared to the model control group. In contrast, the model siNEAT1 group showed a decrease in NMDA receptor expression (P = 0.0304) and an increase in GABA‐A receptor expression (P = 0.0003) compared to the model control group, n = 3. I‐K): In the acquisition trial, compared to the model NEAT1 group, the model siNEAT1 group exhibited a significantly shorter path length when navigating from the farthest quadrant (4th and 7th) to the target quadrant on the second and third training days, with statistically significant differences observed on the fourth training session. Additionally, in terms of average latency, the model NEAT1 group demonstrated a longer latency during the fourth training session compared to the model control group. No significant differences in swimming speed were observed between groups, n = 6. L): In the probe trial, after removing the platform, the number of times mice in the model control group crossed the platform site was significantly lower than that in the control group (P = 0.0018). The model siNEAT1 group exhibited a significantly higher number of platform crossings compared to both the model NEAT1 group (P < 0.0001) and the model control group (P = 0.0032), n = 6. M‐N): In trial 1, mice in the model siNEAT1 group showed a gradual reduction in the time required to locate the new platform during training on the second (P = 0.0147) and third training days (P = 0.0288). Similarly, in trial 2, due to enhanced memory of the previous platform location, the model siNEAT1 group demonstrated a progressive reduction in search time on the second (P = 0.0003), third (P = 0.0014), and fourth (P = 0.0006) training days, n = 6. O): In the probe trial, the swimming routes of mice in each group within 60 s were recorded and analyzed. Statistical analyses were carried out via one‐way ANOVA for (A, F‐H), and two‐way ANOVA for (I‐L) with the Bonferroni test. ^*^ *p* < 0.05, ^**^ *p* < 0.01, ^***^ *p* < 0.001, and ^****^ *p* < 0.0001.

To evaluate the impact of NEAT1 on seizures, we analyzed seizure behavioral assessments alongside EEG recordings in a mouse model. We conducted a statistical analysis of seizure frequency distribution among individual mice in the TSC model during the preliminary experiments and observed no significant inter‐group differences in variability. Mice in the model control group exhibited a significantly higher seizure frequency compared to the control group (*P* < 0.0001) (**Figure** **3**A–C). The seizure frequency of mice in model NEAT1 group was significantly higher than that in model control group (*P* = 0.0002) (Figure 3A,C,D), while the model siNEAT1 group showed a significant reduction in seizure frequency (*P* < 0.0001) (Figure 3A,C and 3E). Furthermore, we investigated whether an imbalance in neurotransmitter receptor expression might contribute to the seizures mediated by NEAT1. The expression of NMDA receptors (*P* < 0.0001) was higher in TSC2‐CKO mice compared to wild‐type mice, while GABA‐A receptor (*P* < 0.0001) expression was reduced (Figure 3F–H). In the model NEAT1 group, NMDA receptor expression was significantly elevated (*P* = 0.0017), while GABA‐A receptor expression was reduced (*P* = 0.0026) compared to the model control group (Figure 3F–H; Figure S1A, Supporting Information). In contrast, the model siNEAT1 group showed a decrease in NMDA receptor expression (*P* = 0.0304) and an increase in GABA‐A receptor expression (*P* = 0.0003) compared to the model control group (Figure 3F–H; Figure S1A, Supporting Information).

### 3.3 NEAT1 Regulates Cognition and Memory in Animal Model

In the acquisition trial, no significant differences in swimming speed were observed between groups, indicating that general motor function was not impaired and unlikely to affect spatial learning performance (Figure 3K). However, compared to the model NEAT1 group, the model siNEAT1 group exhibited a significantly shorter path length when navigating from the farthest quadrant (4th and 7th) to the target quadrant on the second and third training days, with statistically significant differences observed on the fourth training session (Figure 3I). Additionally, in terms of average latency, the model NEAT1 group demonstrated a longer latency during the fourth training session compared to the model control group (Figure 3J).

In the probe trial, after removing the platform, the number of times mice in the model control group crossed the platform site was significantly lower than that in the control group (*P* = 0.0018) (Figure 3L). Moreover, the model siNEAT1 group exhibited a significantly higher number of platform crossings compared to both the model NEAT1 group (*P* < 0.0001) and the model control group (P = 0.0032) (Figure 3L). Furthermore, in the probe trial, the percentage of time spent in the target quadrant was significantly increased in the model siNEAT1 group compared to the model NEAT1 group (*P* = 0.0436) (Figure 3O; Figure S1B, Supporting Information).

To assess the working memory and short‐term memory of the TSC2‐CKO model of mice, the matching‐to‐sample test in the Morris water maze was used. No significant differences in the average latency were observed between trials 1 and 2 for each group. In trial 1, mice in the model siNEAT1 group showed a gradual reduction in the time required to locate the new platform during training on the second (*P* = 0.0147) and third training days (*P* = 0.0288) (Figure 3M). Similarly, in trial 2, due to enhanced memory of the previous platform location, the model siNEAT1 group demonstrated a progressive reduction in search time on the second (*P* = 0.0003), third (*P* = 0.0014), and fourth (*P* = 0.0006) training days (Figure 3N). This trend was consistently observed across both trial 1 and trial 2.

### 3.4 NEAT1 Regulates PI3K/AKT/mTOR Signaling Pathway

**Figure 4:**
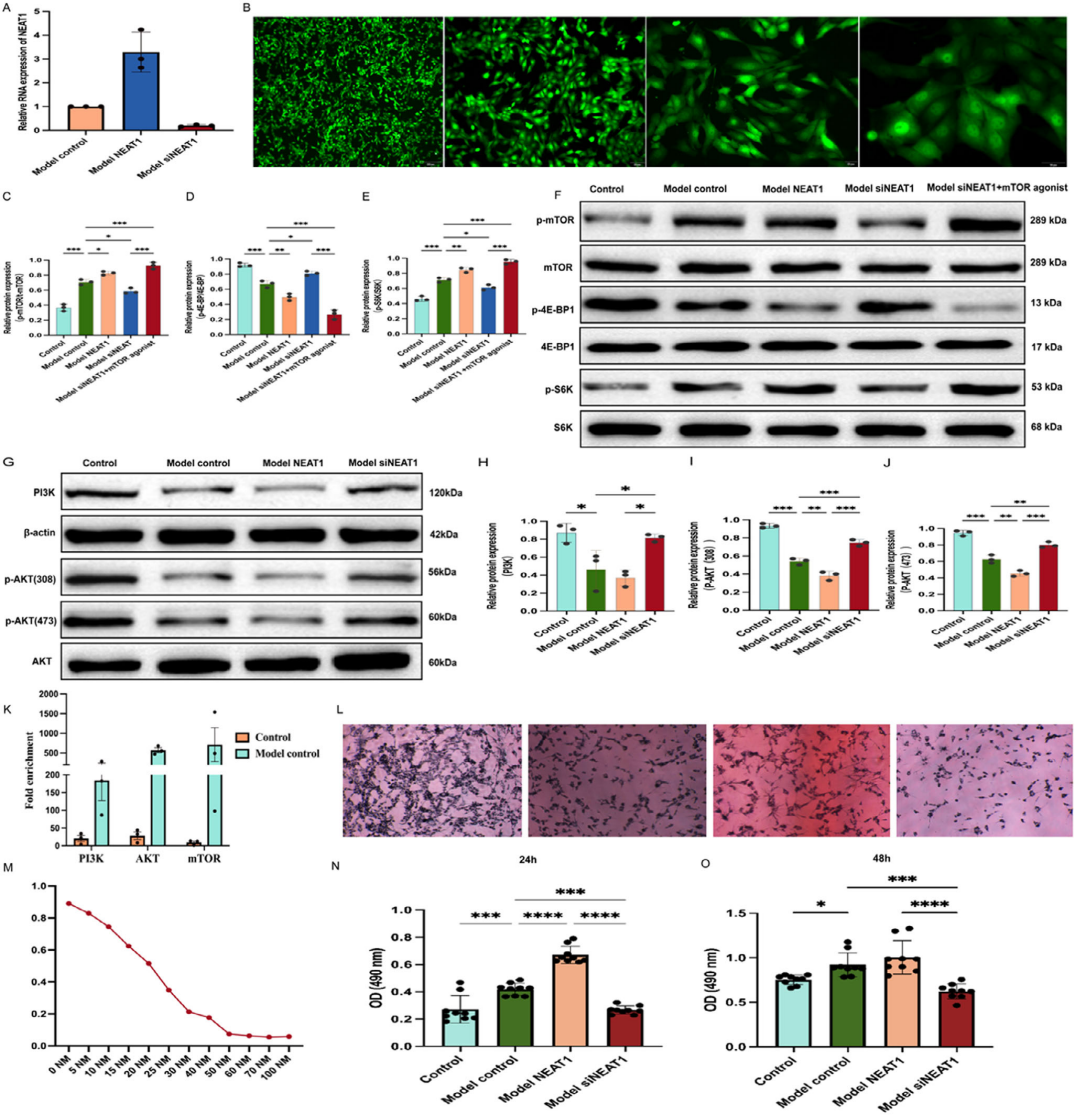
LncRNA NEAT1 regulate downstream pathway targeting mTOR. A) qPCR analysis to verify the transfection efficiency of NEAT1 lentivirus. Compared to the model control, model NEAT1 showed significant overexpression, while model siNEAT1 exhibited significant interference, n = 3. B) Stable transfection of siNEAT1 lentivirus in cells showed green fluorescence, observed under a fluorescence microscope at 4x, 10x, 20x, and 40x magnifications (GFAP staining). C‐F) Compared to the control group, the model control group exhibited significantly decreased p4E‐BP1 phosphorylation alongside increased p‐mTOR and p‐S6K1 levels. In contrast, the model siNEAT1 group exhibited significantly upregulated p4E‐BP1 phosphorylation, accompanied by a reduction in p‐mTOR and p‐S6K1 levels. mTOR agonist was added in the siNEAT1 group successfully reversed the observed changes in the phosphorylation of downstream signaling proteins, n = 3. G‐J) The expression of PI3K and phosphorylated AKT (Thr308) was downregulated in both the model control and model NEAT1 groups, while it was significantly downregulated in the control and model siNEAT1 groups. Additionally, the phosphorylation site of AKT at ser473, representing mTORC2, was also downregulated in the model control and model NEAT1 groups, n = 3. K) NEAT1 was significantly enriched in the brain tissue of model control (TSC2‐CKO) group in RIP experiment. L)After adding 0, 20, 25, and 50 nm concentrations of rapamycin to TSC2‐KO cells for 24 h, the MTT assay was performed, and the results were observed under a 10x objective lens using an optical microscope. M) After adding different concentrations of rapamycin to TSC2‐KO cells for 24 h, the MTT assay was performed, and the absorbance was measured. The half‐maximal inhibitory concentration of rapamycin was range from 20 to 25 nm. N‐O) After 24 h of growth, compared to the model control group, the absorbance ratio in model NEAT1 group was significantly higher, while the absorbance in the siNEAT1 group was reduced, with cells appearing more sparsely distributed. After 48 h of growth, the siNEAT1 group continued to have the lowest number of surviving cells among all groups. Compared to the model control group, the absorbance ratio in control group was still significantly higher, n = 3. Statistical analyses were carried out via one‐way ANOVA for (C‐J), and two‐way ANOVA for (N‐O) with the Bonferroni test. ^*^ *p* < 0.05, ^**^ *p* < 0.01, ^***^ *p* < 0.001, and ^****^ *p* < 0.0001.

RNA sequencing revealed that differentially expressed genes in ETs were enriched in the PI3K‐AKT pathway, and the PI3K/AKT/mTOR pathway is known to be the pathological basis of TSC‐related epilepsy (Figure 2D). To investigate the role of NEAT1 in this pathway, we successfully overexpressed and silenced NEAT1 in TSC2‐KO cell model for functional validation (**Figure** **4**A,B). Given that mTOR, 4E‐BP1, and S6K exert their effects through phosphorylation, we examined their phosphorylation levels relative to total protein. While the total protein levels of mTOR, S6K1, and 4E‐BP1 remained unchanged across groups, distinct phosphorylation patterns were observed. Compared to the control group, the model control group exhibited significantly decreased p4E‐BP1 phosphorylation alongside increased p‐mTOR and p‐S6K1 levels (Figure 4C–F). In contrast, the model siNEAT1 group exhibited upregulated p4E‐BP1 phosphorylation, accompanied by a reduction in p‐mTOR and p‐S6K1 levels (Figure 4C–F). The model NEAT1 group exhibited the opposite pattern, reinforcing the regulatory role of NEAT1 in this pathway. Notably, treatment with an mTOR agonist in the siNEAT1 group successfully reversed the observed changes in the phosphorylation of downstream signaling proteins (Figure 4C–F). To further elucidate the molecular mechanisms involved, we assessed PI3K and AKT expression. In the model NEAT1 group, total PI3K levels, as well as p‐AKT (Thr308), were significantly reduced (Figure 4G–J). Additionally, p‐AKT (Ser473) was also markedly changed among the four groups (Figure 4G‐J).

In addition, we performed RNA immunoprecipitation(RIP) assays on brain tissues from wild‐type and TSC2‐KO mice. The results showed that NEAT1 was significantly enriched in complexes with PI3K, AKT, and mTOR in the TSC2‐KO group, indicating a direct molecular interaction between NEAT1 and these components (Figure 4K).

### 3.5 NEAT1 Regulates Cell Survival and Proliferation

To investigate the role of NEAT1 in cell survival and proliferation, MTT assay was conducted. After 24 h of growth, compared to the model control group, the absorbance ratio in model NEAT1 group was significantly higher, while the absorbance in the siNEAT1 group was reduced, with cells appearing more sparsely distributed (Figure 4N). After 48 h of growth, the siNEAT1 group continued to have the lowest number of surviving cells among all groups (Figure 4O). To further examine the involvement of the mTOR pathway, we determined the half‐maximal inhibitory concentration (IC50) of an mTOR inhibitor‐rapamycin, which ranged from 20 to 25 nm (Figure 4L,M). Based on this, we treated the control, model control, model NEAT1, and model siNEAT1 groups with 25 nm of the mTOR inhibitor and compared them to their respective untreated counterparts. The mTOR inhibition effectively suppressed cell proliferation after both 24 and 48 h of treatment, with the most pronounced inhibitory effect observed in the siNEAT1 group (*P*<0.0001)(Figure S1C‐1D, Supporting Information).

### 3.6 NEAT1 Regulates Neurotic Electrophysiology‐ Potassium Currents

**Figure 5:**
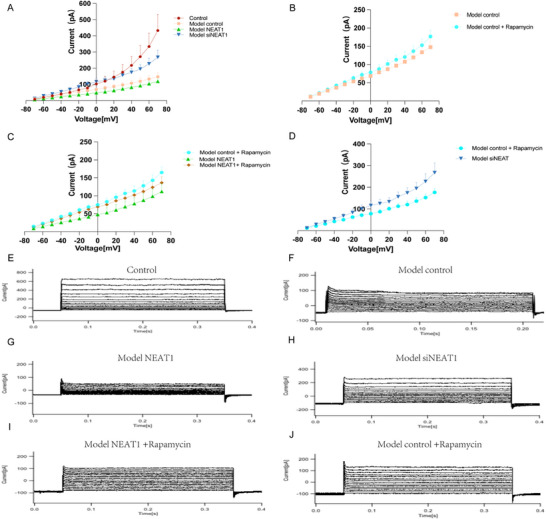
LncRNA NEAT1 mediates outward potassium current. A) The outward potassium ion current is most significant in the control group (433.00 ±199.50). The model siNEAT1 group exhibited significantly increased outward potassium current compared to model control group (267.70 ± 89.87 vs 147.50 ± 47.41 at 70 mV, P = 0.0103), whereas the model NEAT1 group showed the lowest outward K^+^ current, n = 3. B) The addition of rapamycin to the model control group did not result in any changes in potassium ion current, n = 3. C) The addition of rapamycin to the model NEAT1 group did not result in any changes in potassium ion current. The addition of rapamycin to the model control group resulted in a higher level of potassium ion efflux compared to the model NEAT1 group, n = 3. D) The outward K+ current in the model siNEAT1 group was significantly higher than that in model control + Rapamycin group (267.70 ± 89.87 vs 176.70 ± 34.84, P = 0.0047), n = 3. E‐J) The current‐voltage performance from ‐70 to 70 mV, with a 10 mV step interval, for the following groups: control, model control, model NEAT1, model siNEAT1, model control + Rapamycin, and model NEAT1 + Rapamycin. Statistical analyses were carried out via one‐way ANOVA with the Bonferroni test for (A‐D). ^*^ *p* < 0.05, ^**^ *p* < 0.01, ^***^ *p* < 0.001, and ^****^ *p* < 0.0001.

To validate the regulatory role of NEAT1 in ion currents, we performed patch‐clamp recordings on cells from four different groups. The cells were voltage‐clamped at −70 mV and stepped from ‐70 to 70 in 10 mV increments. Among the groups, the model siNEAT1 group exhibited significantly increased outward potassium (K+) current compared to model control group (267.70 ± 89.87 vs 147.50 ± 47.41 at 70 mV, P = 0.0103), whereas the model NEAT1 group showed the lowest outward K+ current (**Figure** **5**A and Figure 5E–H). To further investigate the involvement of the mTOR pathway, the mTOR inhibitor‐rapamycin was applied on model control and model NEAT1 group to assess its effect in K+ current. The mTOR inhibitor did not significantly restore the outward current inhibition observed in the model NEAT1 group and model control group (Figure 5B,C and Figure 5I). However, the outward K+ current in model control + Rapamycin group remained significantly higher than that in the model NEAT1 group (176.70 ± 34.84 vs 118.80 ± 48.59, P = 0.0037) (Figure 5C). Moreover, the outward K+ current in the model siNEAT1 group was significantly higher than that in model control + Rapamycin group (267.70 ± 89.87 versus 176.70 ± 34.84, P = 0.0047) (Figure 5D,J).

## 4 Discussion

TSC represents a prototypic monogenic disorder of the mTOR pathway dysregulation establishing mechanistic basis of a direct link between genetic variants and brain pathology underlying intractable epilepsy.^[^ ^2^ ^]^ Several studies have addressed the cellular and molecular pathways that contribute to epileptogenesis, with the aim to develop targeted therapies to effectively prevent or halt this process.^[^ ^1^ ^]^ However, the clinical efficacy of rapamycin has been limited. Therefore, we explored the role of epigenetics in TSC‐associated epilepsy.

We conducted lncRNA genomic analysis on clinical specimens and verified the differential NEAT1 expression in epileptogenic and non‐epileptogenic tubers, establishing a foundation for our experiment. NEAT1 was predominantly highly expressed in the cerebral cortex and could regulate neuronal cell differentiation, proliferation, as well as the PI3K‐AKT‐mTOR signaling pathway.^[^ ^2^, ^9^ ^]^ Furthermore, enrichment analysis verified that upregulated genes were enriched in the PI3K‐AKT pathway and other immune‐inflammation pathways.^[^ ^9^ ^]^ These findings underscore the intrinsic tissue pathology associated with epileptic seizures in TSC and highlight the importance of NEAT1 and PI3K/AKT/mTOR pathway.

TSC is most notably associated with two disabling manifestations: intractable epilepsy and intellectual disability.^[^ ^1^, ^23^ ^]^ To further investigate the expression and role of NEAT1 in TSC‐related epilepsy and cognitive function, we utilized the TSC2‐CKO mouse model to create NEAT1 overexpression and interference models. Our findings revealed that reduced expression of NEAT1 alleviated epileptic seizures, consistent with previous research indicating that NEAT1 can modulate seizure activity.^[^ ^24^ ^]^ Specifically, NEAT1 influences seizure activity through regulating neuronal function and inflammatory responses.^[^ ^22^, ^24^ ^]^ This hyperactivation is closely linked to the pathophysiology of TSC‐related epilepsy. In addition, a key aspect of TSC‐related epilepsy is the interaction between GABAergic signaling and mTOR dysregulation. Previous studies have demonstrated delayed or persistently impaired GABAergic maturation, leading to an imbalance between excitatory and inhibitory neuronal networks.^[^ ^8^ ^]^ An imbalance in NMDA and GABA receptor expression in both overexpressed NEAT1 and the TSC2‐CKO mice was observed in this study. In the context of TSC‐related epilepsy, low NEAT1 expression appears to mitigate seizure activity, suggesting that NEAT1 may contribute to epilepsy pathogenesis by disrupting neurotransmitter receptor balance to modulate seizure activity. Furthermore, we observed that TSC2‐CKO mice with NEAT1 interference exhibited improved spatial cognition when exploring the platform. These mice also demonstrated enhanced learning ability and working memory during the matching‐to‐sample training task. This cognitive improvement supports the notion that modulating NEAT1 expression could have therapeutic potential, not only in controlling epileptic seizures but also in enhancing cognitive function. Overall, these findings highlight NEAT1 as a potential therapeutic target for addressing both seizure activity and cognitive impairments in TSC.

TSC establishes a mechanistic link between genetic mutations and the complex clinical phenotype of intractable epilepsy.^[^ ^7^, ^8^ ^]^ A key pathological feature of epileptogenesis in TSC is impaired potassium ion dynamics.^[^ ^29^, ^30^ ^]^ While giant cells in TSC cannot generate action potentials, they may buffer potassium accumulation, potentially modulating neuronal excitability.^[^ ^11^, ^17^, ^30^ ^]^ In this study, The outward current in the siNEAT1 group was stronger than in the other two model groups, and the addition of the mTOR inhibitor did not alter the outward potassium current. Our findings provide evidence that NEAT1 regulates outward potassium currents, promoting hyperpolarization and influencing neuronal excitability. However, the specific potassium channel subtypes remain unidentified. This highlights the role of NEAT1 in the electrophysiological and molecular mechanisms underlying TSC‐related epilepsy. Additionally, functional enrichment research has demonstrated that NEAT1 was highly expressed in the cerebral cortex and played a role in regulating multiple inflammatory signaling pathways, including the PI3K‐AKT‐mTOR pathway. The mTOR signaling pathway operates through mTORC1 and mTORC2 complexes, which differ in their molecular composition and sensitivity to rapamycin. mTORC1 is acutely inhibited by rapamycin, whereas mTORC2 is insensitive to rapamycin.^[^ ^4^, ^11^, ^12^ ^]^ TSC‐related phenotypes have traditionally been attributed to mTORC1 hyperactivation, and NEAT1 influences seizures by regulating downstream pathways through mTORC1.^[^ ^12^ ^]^ NEAT1 may serve as an upstream epigenetic modulator of the PI3K/AKT/mTOR axis, thus integrating into the core TSC signaling cascade. This mechanistic link appears to be more pronounced or unique in the context of TSC, where baseline mTOR activity is already dysregulated. This dual regulatory capacity may represent a TSC‐specific mechanism, as such combined effects on both mTOR‐driven cell signaling and electrophysiological excitability have not been reported in other epilepsy subtypes, such as temporal lobe epilepsy, where mTOR signaling remains largely intact. Therefore, NEAT1 may act as an epigenetic amplifier of the mTOR pathway in the unique context of TSC, while simultaneously contributing to circuit‐level dysfunction, providing mechanistic insight and potential therapeutic relevance specific to this disorder.^[^ ^31^, ^32^ ^]^ Our results demonstrated that alterations in NEAT1 significantly impacted the expression of mTOR and downstream proteins (4E‐BP1 and S6K). Additionally, NEAT1 changes also influenced upstream PI3K and AKT signaling, likely due to a negative feedback effect caused by downstream signaling. Furthermore, RIP assays confirmed a direct interaction between NEAT1 and key components of the PI3K‐AKT‐mTOR signaling pathway, suggesting that NEAT1 may regulate the function or stability of these molecules through direct binding and exert regulatory effects at the post‐transcriptional level. This finding supports the feasibility of NEAT1 as a therapeutic target, as it not only modulates epigenetic regulation but also directly influences the functional status of critical signaling proteins. This study provides evidence that TSC‐related epilepsy involves not only mTORC1 hyperactivity but also epigenetic regulation. These insights enhance the understanding of mTOR pathway involvement in TSC‐related epilepsy and underscore the potential of targeting mTORC1, as well as epigenetic factors like NEAT1, for more effective therapeutic strategies. In addition, AKT(ser473), which is fully phosphorylated by mTORC2, also exhibited changes in response to NEAT1 alterations, indirectly indicating the mTORC2 involvement, which is a preliminary step.

### 4.1 Limitations

This study has some limitations. Due to the rarity of this neurological syndrome, the clinical sample size remains insufficient, and further collection of larger samples is necessary for more comprehensive and in‐depth analysis. Due to the ethical constraints, it is not feasible to remove non‐ETs from distant brain regions solely for research purposes. Therefore, we carefully selected patients who presented with multiple tubers in the same lobe or adjacent regions, and where intracranial stereo‐EEG recordings allowed precise differentiation between ETs and non‐ETs. The inclusion criteria are stringent but necessary to ensure accurate comparison while minimizing unnecessary tissue removal. This research has conducted only preliminary investigations into mTORC2, and the underlying mechanisms require further exploration. Additionally, in various potassium ion channel subtypes, we have not yet identified a representative subtype. This study did not perform pharmacological dissection using selective K⁺ channel blockers to pinpoint the affected channel types, which was a priority for future electrophysiological studies. To explore potential interactions between lncRNAs and microRNAs, we preliminarily analyzed differentially expressed miRNAs between epileptogenic and non‐epileptogenic tubers and compared them with miRNA profiles from NEAT1‐overexpression and knockdown cell models. However, due to biological heterogeneity between tissue and cellular systems, no consistently overlapping candidate miRNAs were identified across both datasets. Future studies are needed to refine the disease‐model consistency and explore this mechanism in greater depth.

### 4.2 Future Directions

Targeting NEAT1 with antisense oligonucleotides or siRNAs, in combination with established mTOR inhibitors such as everolimus, represents a promising dual‐approach strategy for the treatment of TSC‐related epilepsy. This method may synergistically address both upstream epigenetic dysregulation and downstream mTOR pathway hyperactivation, potentially improving seizure control, reducing drug dosages, and minimizing side effects. Preclinical studies have demonstrated the feasibility of RNA‐based therapeutics in central nervous system disorders, particularly when coupled with advanced nanotechnology‐enabled delivery systems.^[^ ^33^ ^]^ However, clinical translation of NEAT1‐targeted therapies faces several hurdles, including the lack of validated brain‐specific delivery systems, risk of off‐target effects in non‐neuronal tissues, and insufficient data on pharmacokinetics, biodistribution, and long‐term safety. Future studies should aim to clarify the upstream regulatory mechanisms of NEAT1, characterize its dose‐response and off‐target profiles, develop CNS‐penetrant delivery vectors with cell‐type specificity, and conduct comprehensive safety assessments—covering immunogenicity, tolerability, and behavioral outcomes. These efforts are essential to establish NEAT1 as a viable therapeutic target in the evolving treatment paradigm of TSC‐associated epilepsy.

## 5 Conclusions 

This study provides novel insights into the epigenetic regulation of TSC‐associated epilepsy, demonstrating that NEAT1 contributes to both mTORC1‐ and mTORC2‐dependent mechanisms. NEAT1 influences epileptic seizures through the regulation of the PI3K/AKT/mTOR pathway, neurotransmitter balance, and outward potassium currents, involving three key aspects: inflammatory responses, neurotransmitter receptors, and potassium ion channels. Additionally, the differential expression of NEAT1 also has a notable impact on spatial memory and working memory. The involvement of mTORC2, mediated by NEAT1, provides further insights into the complexity of TSC epileptogenesis. Future research should further investigate NEAT1's therapeutic potential and its role in stratifying epilepsy phenotypes for personalized treatment approaches.

## Conflict of Interest

The authors declare no conflict of interest.

## Ethics Approval and Consent to Participate

This study was approved by the Ethics Committee of Beijing Children's Hospital, Capital Medical University ([2022]‐E‐021‐Y; 2022‐01‐25), and written informed consent was obtained from each patient. Care and use of mice were conducted according to an animal protocol approved by SPF (Beijing) Biotechnology Co., Ltd (AWE 2023040401; 2023‐04‐04).

## Author Contributions

Conceptualization: S.K. and S.L. Resource: Z.W. and J.H. Software: J.W. and T.L. Methodology: S.K. and S.L. Validation: Z.W. and T.L. Investigation: Y.T., F.Z., and J.X. Funding acquisition: S.L. Writing‐original draft: S.K. and S.L. Writing‐review and editing: T.L., Z.W., J.H., J.X., J.W., F.Z., and Y.T. All authors approved the final version of the manuscript.

## Supporting information



Supporting Information

## Data Availability

The data that support the findings of this study are available from the corresponding author upon reasonable request.
