# Supplementary material for: NEAT1 Promotes Epileptogenesis in Tuberous Sclerosis Complex
Source: Adv Sci (Weinh). 2025 Oct 15;13(1):e04316. doi: 10.1002/advs.202504316 (PMC12767121; doi:10.1002/advs.202504316)
Supplement: Supplementary file 1 — Supporting Information [file ADVS-13-e04316-s001.docx]

**Supplementary Figure 1**

**
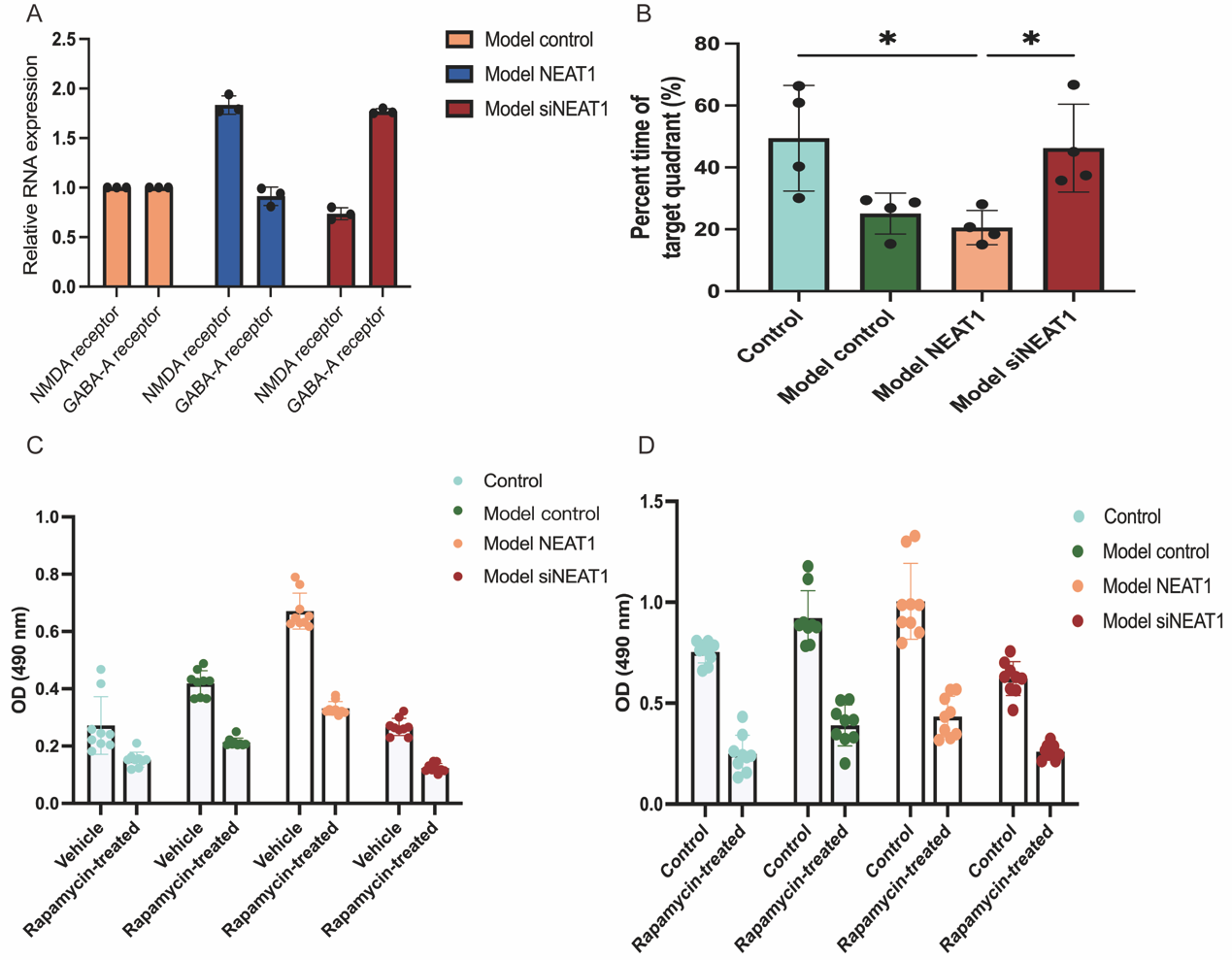
**

**Supplementary Figure 1.** The role of NEAT1 in neuronal excitability, behavior, and proliferation.

Legend: A) In the detection of NMDA receptors and GABA receptors in the mouse cortex, the model NEAT1 group showed overexpression of *NMDA receptor* gene and decreased expression of *GABA receptor*, while the model siNEAT1 group showed the opposite results, n=3. B) In the probe trial, the percentage of time spent in the target quadrant was significantly increased in the Model siNEAT1 group compared to the Model NEAT1 group (P=0.0436), n=6. C-D) The control, model control, model NEAT1, and model siNEAT1 groups were treated with 25 nM of the rapamycin and compared them to their respective untreated counterparts. The mTOR inhibition effectively suppressed cell proliferation after both 24 and 48 hours of treatment, with the most pronounced inhibitory effect observed in the siNEAT1 group (P<0.0001), n=3. Statistical analyses were carried out via one-way ANOVA with the Bonferroni test for (A-D). *p < 0.05, **p < 0.01, ***p < 0.001, and ****p < 0.0001.
